# The IRE1α Inhibitor KIRA6 Blocks Leukotriene Biosynthesis in Human Phagocytes

**DOI:** 10.3389/fphar.2022.806240

**Published:** 2022-03-22

**Authors:** Xiao Tang, Tarvi Teder, Bengt Samuelsson, Jesper Z. Haeggström

**Affiliations:** Division of Physiological Chemistry II, Department of Medical Biochemistry and Biophysics, Karolinska Institutet, Stockholm, Sweden

**Keywords:** leukotrienes, KIRA6, inflammation, IRE1α, MAPKs

## Abstract

The ER stress and Unfolded Protein Response (UPR) component inositol-requiring enzyme 1α (IRE1α) has been linked to inflammation and lipid mediator production. Here we report that the potent IRE1α inhibitor, KIRA6, blocks leukotriene biosynthesis in human phagocytes activated with lipopolysaccharide (LPS) plus N-formyl-methionyl-leucyl-phenylalanine (fMLP) or thapsigargin (Tg). The inhibition affects both leukotriene B_4_ (LTB_4_) and cysteinyl leukotriene (cys-LTs) production at submicromolar concentration. Macrophages made deficient of IRE1α were still sensitive to KIRA6 thus demonstrating that the compound’s effect on leukotriene production is IRE1α-independent. KIRA6 did not exhibit any direct inhibitory effect on key enzymes in the leukotriene pathway, as assessed by phospholipase A_2_ (PLA_2_), 5-lipoxygenase (5-LOX), LTA_4_ hydrolase (LTA4H), and LTC_4_ synthase (LTC4S) enzyme activity measurements in cell lysates. However, we find that KIRA6 dose-dependently blocks phosphorylation of p38 and ERK, mitogen-activated protein kinases (MAPKs) that have established roles in activating cytosolic PLA_2_α (cPLA_2_α) and 5-LOX. The reduction of p38 and ERK phosphorylation is associated with a decrease in cPLA_2_α phosphorylation and attenuated leukotriene production. Furthermore, KIRA6 inhibits p38 activity, and molecular modelling indicates that it can directly interact with the ATP-binding pocket of p38. This potent and unexpected, non-canonical effect of KIRA6 on p38 and ERK MAPKs and leukotriene biosynthesis may account for some of the immune-modulating properties of this widely used IRE1α inhibitor.

## Introduction

Leukotrienes (LTs) are a class of lipid mediators derived from arachidonic acid (AA), predominantly produced in myeloid cells (granulocytes, monocytes/macrophages and mast cells). There are two types of leukotrienes-leukotriene B_4_ (LTB_4_), a potent chemoattractant and pro-inflammatory mediator, and cysteinyl leukotrienes (cys-LTs, which comprise LTC_4_, D_4_, and E_4_), referred to as slow-reacting substance of anaphylaxis (SRS-A) (Samuelsson, 1983). These mediators act in an autocrine or paracrine manner, exerting their functions through their cognate G-protein coupled receptors (GPCRs) on the target cells (Haeggström and Funk, 2011). The biosynthesis of leukotrienes requires several enzymatic steps. First, phospholipase A_2_ (PLA_2_), especially cytosolic PLA_2_α (cPLA_2_α), catalyzes the release of free AA from membrane phospholipids, which is believed to be the rate-limiting step for the cellular output of leukotrienes and prostaglandins (PGs) (Funk, 2001). In a second step, the key enzyme 5-lipoxygenase (5-LOX) converts free AA to the transient epoxide intermediate LTA_4_ which, depending on the availability of downstream enzymes, is rapidly converted to LTB_4_ by LTA_4_ hydrolase (LTA4H), or to LTC_4_ by LTC_4_ synthase (LTC4S). Together with the accessory proteins in the pathway, these enzymes form the leukotriene biosynthetic complex and control cellular production of leukotrienes (Haeggström, 2018).

In phagocytes, the activities of cPLA_2_α and 5-LOX are regulated by cytosolic calcium and protein phosphorylation (Gijón et al., 2000; Rådmark et al., 2007). In resting cells, cPLA_2_α and 5-LOX are located in the cytosol, or in some cell types, in a nuclear soluble compartment associated with chromatin (Rådmark et al., 2007). Classical leukotriene stimuli (e.g., PAF, fMLP, thapsigargin, and A23187) cause calcium mobilization to the cytosol and binding to the C2 or C2-like domain of cPLA_2_α and 5-LOX, respectively. Simultaneously, cPLA_2_α and 5-LOX are phosphorylated by certain mitogen-activated protein kinases (e.g., p38 and ERK), which also modulates the activity of the two enzymes (Werz, 2002).

At the center of the most conventional arm of unfolded protein response (UPR), inositol-requiring enzyme 1α (IRE1α) plays an important role in sensing misfolded proteins and maintaining homeostasis of the endoplasmic reticulum (ER) (Hetz, 2012). Exposure of misfolded proteins leads to autophosphorylation of IRE1α at its kinase domain and oligomerization, which in turn causes activation of its RNase domain, resulting in splicing and activation of the transcription factor XBP1 (Korovesis et al., 2020). However, hyperactive IRE1α degrades ER-localized RNA and cause cell death (Ghosh et al., 2014). In addition, IRE1α has also been strongly implicated in macrophage polarization, cytokine secretion and prostaglandin production, in several immunological and metabolic disorders (Shan et al., 2017; Smith, 2018; Chopra et al., 2019; Batista et al., 2020; Hull-Ryde et al., 2021).

Small molecules known as kinase-inhibiting RNase-attenuators (KIRA) s have been designed to allosterically inhibit the RNase activity of IRE1α by stabilizing the ATP-binding kinase domain and preventing oligomerization (Ghosh et al., 2014). As an optimized KIRA, KIRA6 has been widely used experimentally to target IRE1α. It has been shown that KIRA6 effectively blocks PGE_2_-mediated pain with a comparable efficacy as celecoxib (Chopra et al., 2019), whereas the effect of KIRA6 and IRE1α activation in leukotriene production remains to be assessed.

## Methods

### Reagents

KIRA6 was from Cayman Chemicals (Ann Arbor, MI, United States). Monoclonal antibodies (mAb) against phosphorylated cPLA_2_α (p-cPLA_2_α) (Ser-505), p-p38 (Thr180/Tyr182), p-ERK (Thr202/Tyr204), and p-5-LOX (Ser271 and Ser663) were from Cell Signaling Technology (Danvers, MA, United States). LPS, Thapsigargin, fMLP 4μ8C, RPMI-1640 cell culture medium, HEPES solution, 1,25-dihydroxy vitamin D3, horseradish peroxidase (HRP)-linked mAb against β-actin were from Sigma-Aldrich (St. Louis, MO, United States). TGF-β1 and M-CSF were from Thermofisher Scientific (Waltham, MA, United States). SB203580 was from Tocris Bioscience (Bristol, UK).

### Cell Culture

Human monocyte-derived macrophages (hMDMs) and neutrophils were isolated as previously described (Wan et al., 2007; Wan et al., 2018). Briefly, peripheral blood monocytes and neutrophils were isolated by dextran sedimentation and gradient centrifugation with Ficoll-paque Premium (GE Healthcare, Little Chalfont, United Kingdom) from freshly prepared buffy coats (Karolinska Blood Bank). Isolated monocytes were further cultured in RPMI-1640 medium supplemented with 10% heat-inactivated fetal bovine serum (FBS) and M-CSF (50 ng/ml) for 6 days to obtain mature macrophages. The human monocytic cell line Mono Mac 6 (MM6) were cultured in RPMI-1640 supplemented with 10% FBS, and differentiated into macrophages with 1,25-dihydroxy vitamin D3 (50 nM) and TGF-β1 (2 ng/ml) for 4 days.

### Generation of Inositol-Requiring Enzyme 1α Knockdown Cells

MM6 cells were transfected with lentiviral shRNA against IRE1α (NM_001433) and non-target shRNA to generate IRE1α knockdown (KD) and control MM6 cells. The knockdown efficiency was evaluated by qPCR analysis of the mRNA expression of IRE1α.

### ELISA

LTB_4_, cys-LTs, and PGE_2_ in cell supernatants were measured with ELISA kits (Cayman chemicals) according to the manufacturer’s instructions. The absorbance at 405 nm was recorded by a TECAN Infinite M200 plate reader.

### Western Blot

Cells were lysed with RIPA buffer supplemented with protease inhibitor and phosphatase inhibitor cocktails (Roche Diagnostics, Mannheim, Germany). SDS-PAGE and western blot transfer were carried out with NuPAGE Novex 4–12% Bis-Tris gels and iBlot2 blotting system (Thermofisher). Afterwards, the membranes were incubated with 3% non-fat milk for 1 h and further incubated with HRP-linked β-actin antibody (Sigma-Aldrich) for 1 h, or desired primary antibodies overnight at 4°C followed by HRP-linked anti-rabbit antibody (GE Healthcare). The enhanced chemiluminescence substrate was then added to the membranes to visualize the immunoreactive protein bands.

### Kinase Activity Assay

The p38 and ERK kinase assays were performed according to the manufacturer’s protocol (Promega) with optimized conditions. The p38 assay was carried out with 10 ng of the p38 enzyme, 150 µM ATP and 0.2 µg/µL p38 peptide as substrates, and 0–10 µM of KIRA6, 4 µ8C or SB203580 as the positive control. The ERK kinase assay was performed with 3 ng of ERK1 or ERK2, 50 µM ATP and 0.5 µg MAP kinase substrate (MBP), together with 0–100 µM of KIRA6 or K252a as the positive control. Produced ADP was converted to ATP by ADP-Glo Assay (Promega) and the luminescence recorded using the TECAN Infinite M200 plate reader. Fluorescence values were normalized to vehicle group and the dose response curve was created using non-linear regression from which IC_50_ values were determined with the GraphPad Prism program.

### Molecular Docking

The crystal structure of p38 in complex with SB203580 (PDB ID: 1A9U) was used as a template to perform docking simulations with KIRA6. KIRA6 as the ligand was prepared using the Grade Web Server (https://www.globalphasing.com). Prior to the docking, SB203580 was removed from the X-ray structure. Docking simulations with KIRA6 was carried out with the AutoDock Vina tool (Trott and Olson, 2010). In parallel, docking with SB203580 was performed to determine the binding score. The binding conformation with the best score was visualized with the Chimera 1.15 software. The 2D map for the protein-ligand interactions were generated using BIOVIA Discovery Studio Visualizer (Dassault Systems) and reproduced using ChemDraw 19.0.

### Statistics

Data were presented as Mean ± SEM unless otherwise specified. Differences among groups were evaluated by Student’s t-test, One-way or Two-way ANOVA. A value of *p* < 0.05 was considered statistically significant.

## Results

### KIRA6 Blocks Leukotriene Production From Macrophages and Neutrophils

**FIGURE 1:**
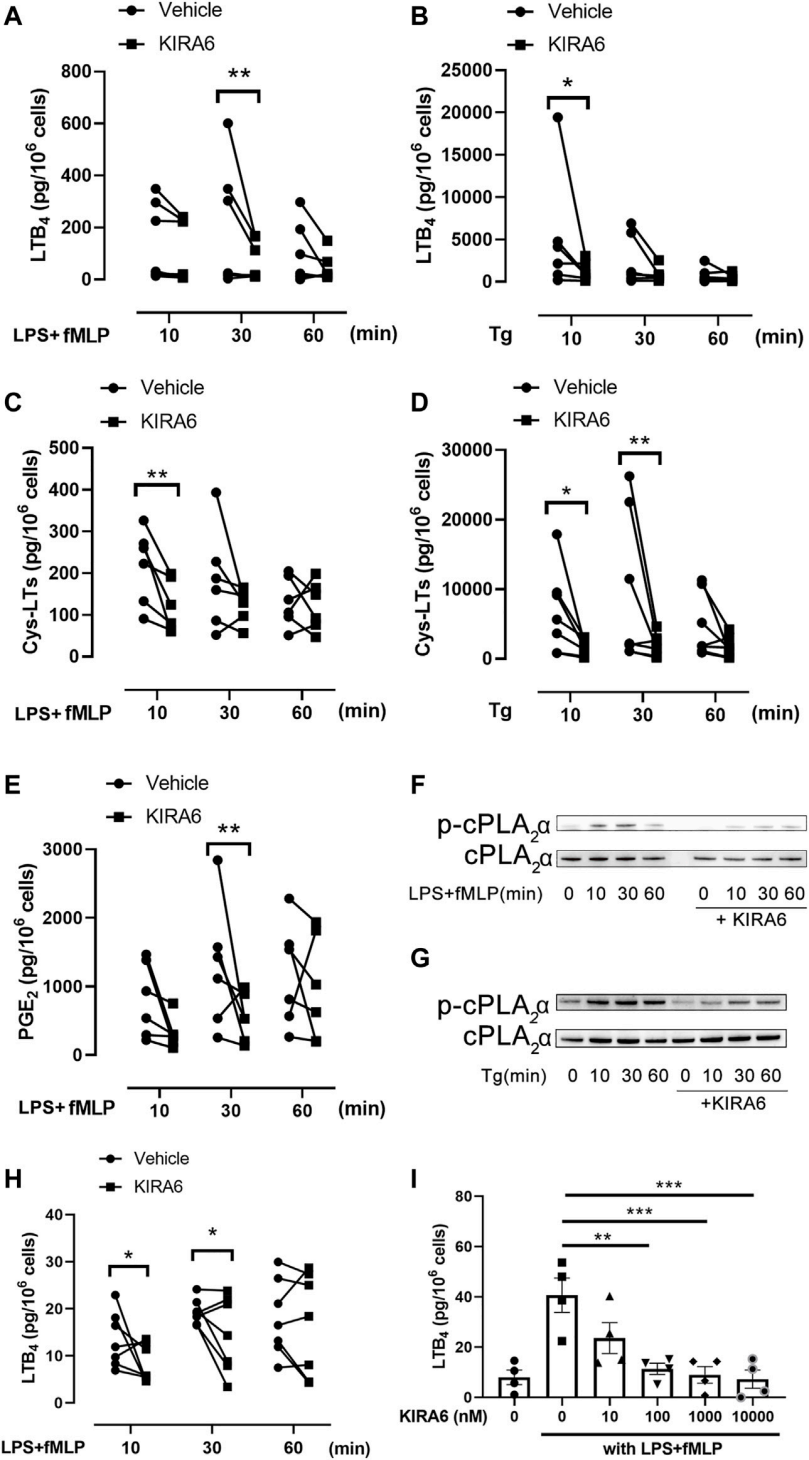
KIRA6 inhibits leukotriene production from hMDMs and neutrophils. **(A–E)** hMDMs were pretreated with 1 µM KIRA6 or vehicle (0.1% DMSO) for 30 min. Afterwards, cells were treated with 100 ng/ml LPS for 10 min, followed by fMLP (1 µM) stimulation for 10, 30 or 60 min **(A,C,E)**, or stimulated with 1 µM thapsigargin (Tg) for the same time **(B,D)**. LTB4, cys-LTs or PGE2 in the cell supernatants were analyzed by ELISA. **(A)**: *n* = 5, **(B–E)**: *n* = 6. **(F,G)** Phosphorylated cPLA_2_α (p-cPLA_2_α) and cPLA_2_α protein were analyzed by western blot (representative of three independent experiments). **(H,I)** Neutrophils were treated with vehicle (0.1% DMSO) or 1 µM KIRA6, followed by LPS (100 ng/ml) and fMLP (1 μM) stimulation for 10, 30 or 60 min **(H)** (*n* = 7). Alternatively, neutrophils were treated with vehicle (0.1% DMSO) or different concentrations of KIRA6 for 30 min, followed by LPS (100 ng/ml, 10 min), and fMLP (1 μM, 30 min) stinulation **(I)** (*n* = 4). LTB_4_ production in cell supernatants was analyzed by ELISA. *p < 0.05, **p < 0.01, ***p < 0.001, ****p < 0.0001.

To assess the effect of KIRA6 on leukotriene biosynthesis, monocytes were isolated from human peripheral blood and further differentiated into macrophages with M-CSF. KIRA6 was added to the cells before stimulation with LPS plus fMLP or thapsigargin, an inducer of ER stress. In both conditions, LTB_4_ and cys-LT production were triggered and peaked at 10 or 30 min 1 µM KIRA6 significantly inhibited the peak production of LTB_4_ and cys-LTs (Figure 1A–D). The inhibition was not limited to leukotrienes and also affected synthesis of prostaglandins (Figure 1E), suggesting an inhibition of AA release. Since cPLA_2_α is the key enzyme for AA release in macrophages, and cPLA_2_α activity is dependent on phosphorylation at Ser-505 (Gijón et al., 2000), we measured cPLA_2_α phosphorylation in KIRA6 treated cells and observed a reduction in phosphorylated cPLA_2_α protein level (Figure 1F,G). We further asked if the inhibition of leukotriene production is a cell-specific effect and performed similar experiments with neutrophils isolated from peripheral blood. In neutrophils, KIRA6 inhibited LTB4 production at 10 and 30 min stimulation by LPS/fMLP (Figure 1H). The inhibitory effect of KIRA6 was dose-dependent and started to be statistically significant from 100 nM (Figure 1I).

### Phosphorylation of p38 and ERK Are Diminished in KIRA6 Treated Cells, Association With Decreased Leukotriene Production and Cytosolic PLA_2_α Phosphorylation

**FIGURE 2:**
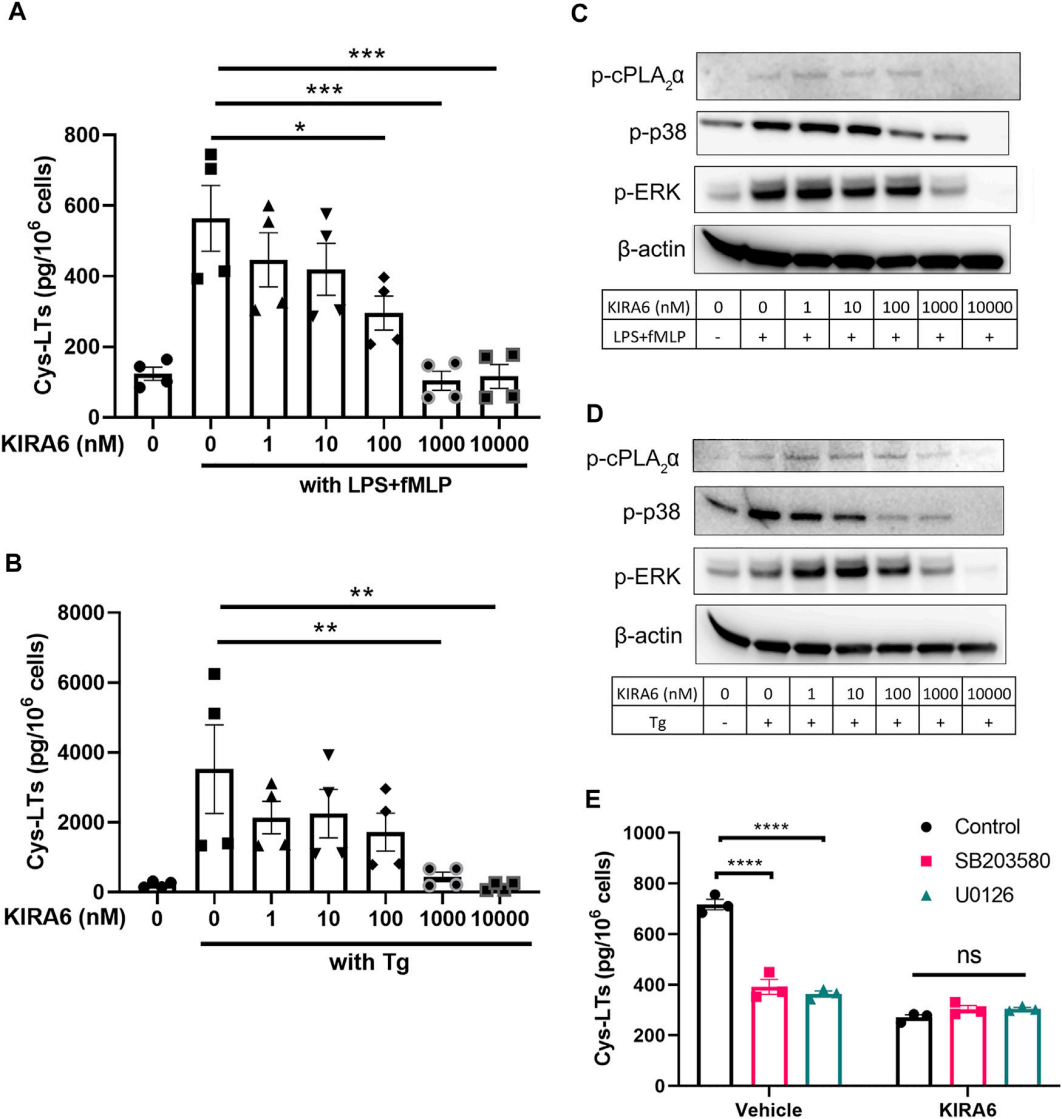
Phosphorylation of p38 and cPLA_2_α is reduced in KIRA6 treated cells **(A,B)**. MM6 cells were pretreated with vehicle (0.1% DMSO) or different concentrations of KIRA6 for 30 min, followed by challenge with LPS (100 ng/ml, 10 min), and fMLP (5 μM, 30 min), or Tg alone (1 μM, 30 min). Cys-LTs in cell supernatants were analyzed by ELISA (*n* = 4). **(C,D)** Phosphorylated cPLA_2_α, p38, and ERK (p-cPLA_2_α, p-p38, and p-ERK) were analyzed by western blot (representative of three independent experiments). **(E)** MM6 cells were pre-treated with vehicle (0.1% DMSO) or KIRA6 (1 µM) followed by incubation of SB203580 (10 µM) or U0126 (10 µM) for 30 min. The cells were further stimulated with LPS (100 ng/ml, 10 min) and fMLP (5 μM, 30 min). Cys-LTs in cell supernatants were analyzed by ELISA (*n* = 3). *p < 0.05, **p < 0.01, ***p < 0.001, ****p < 0.0001, ns: no significance.

To further investigate the mechanisms by which KIRA6 inhibits leukotriene biosynthesis, we measured its effect on enzymatic activity of PLA_2_, 5-LOX, LTA4H, and LTC4S enzyme activity in homogenates of isolated neutrophils and differentiated MM6 cells. KIRA6 failed to attenuate any of these key enzymes in the leukotriene pathway at the concentrations indicated in Supplementary Figure S1. We also tested the effect of KIRA6 on mobilization of cytosolic calcium in hMDMs and found that 1 µM KIRA6 had no significant effect on calcium, also in presence of 2 mM EGTA (Supplementary Figure S2). As cPLA_2_α phosphorylation was inhibited by KIRA6 (Figures 1E,F), we asked whether KIRA6 may target the upstream kinases p38 and ERK. To test this, MM6 cells were used and a similar inhibitory effect of KIRA6 on cys-LT production was observed with IC_50_ values of 89 and 112 nM for the two stimuli, LPS/fMLP and thapsigargin, respectively (Figure 2A,B). KIRA6 dose-dependently inhibited p38 and ERK phosphorylation in MM6 cells, which is associated with attenuation of cPLA_2_α phosphorylation and cys-LT production (Figure 2C,D). KIRA6 inhibited p38 phosphorylation already at 100 nM while a higher concentration (1 µM) was required to inhibit ERK phosphorylation. To further assess if the two kinases are involved in the regulation of cys-LTs by KIRA6, we used the p38 kinase inhibitor SB203580 and U0126- a kinase inhibitor that blocks ERK phosphorylation. As expected, both inhibitors attenuated cys-LTs production from LPS/fMLP stimulated cells. However, they had no apparent inhibitory effects in KIRA6 pre-treated cells, suggesting 1 μM KIRA6 is sufficient to block p38 and ERK pathways to reduce leukotriene production (Figure 2E).

### KIRA6 Inhibits Leukotriene Production Independent of Inositol-Requiring Enzyme 1α

**FIGURE 3:**
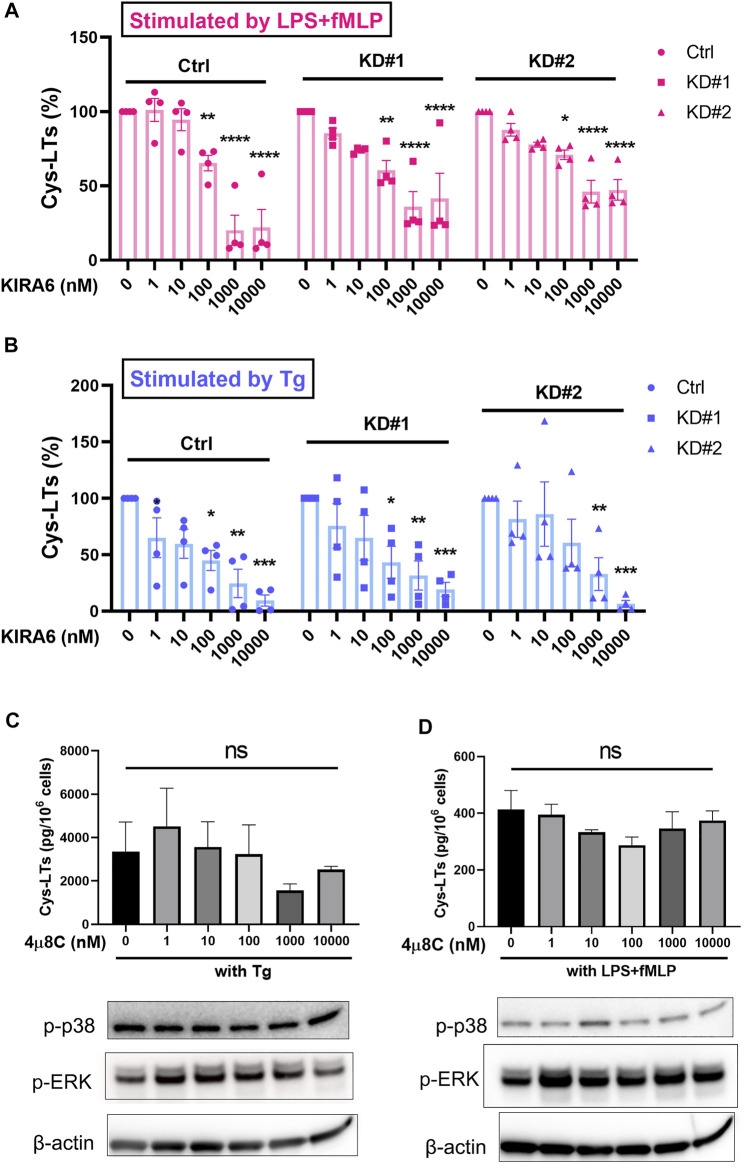
KIRA6 inhibits leukotriene biosynthesis by a mechanism independent of IRE1α. **(A,B)** MM6 cells transfected with shRNA against non-target shRNA (Control) and IRE1α (KD#1 and KD#2) were treated with different concentrations of KIRA6, followed by challenge with LPS (100 ng/ml, 10 min) and fMLP (5 μM, 30 min), or Tg alone (1 μM, 30 min). Cys-LTs production in cell supernatants were analyzed by ELISA (*n* = 4). **(C,D)** MM6 cells were pretreated with vehicle (0.1% DMSO) or different concentrations of 4 µ8C for 30 min, followed by challenge with LPS (100 ng/ml, 10 min) and fMLP (5 μM, 30 min), or Tg alone (1 μM, 30 min). Phosphorylated p38 (p-p38) and p-ERK were analyzed by western blot (representative of three independent experiments). Cys-LT production in cell supernatants were analyzed by ELISA (*n* = 4). *p < 0.05, **p < 0.01, ***p < 0.001, ****p < 0.0001, ns: no significance.

Inasmuch as KIRA6 is a potent IRE1α inhibitor, it was expected that this protein would be involved in the inhibition of leukotriene production. To specifically target IRE1α, we silenced this enzyme in MM6 cells (Supplementary Figure S3) and analyzed the effect of KIRA6. Interestingly, KIRA6 showed significant inhibitory effect on leukotriene production by IRE1α deficient cells, in a similar manner as in control MM6 cells (Figures 3A, B). Furthermore, when control MM6 cells were treated with another potent IRE1α inhibitor, 4 µ8C, the leukotriene production and the phosphorylation of p38 and ERK were not significantly influenced (Figures 3C, D), suggesting the effects of KIRA6 (Figure 2A,B) are IRE1α-independent.

### KIRA6 Is a Potent p38 Inhibitor With Potential Affinity to the ATP-Binding Pocket

**FIGURE 4:**
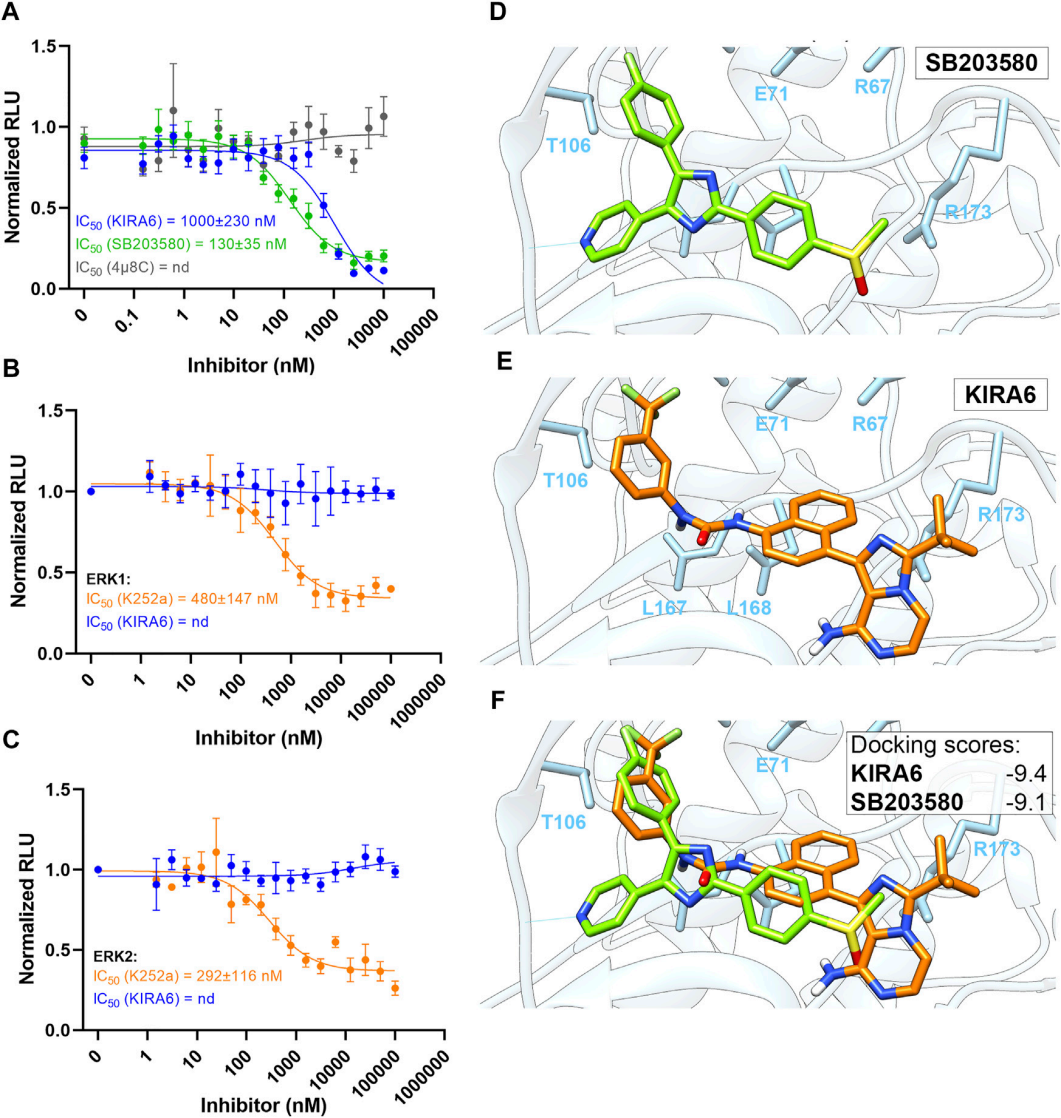
Molecular docking of KIRA6 into the active site of p38. **(A)** Dose response curves of p38 kinase activity inhibition by KIRA6 (blue trace; *n* = 10), SB203580 (green trace; *n* = 4) and 4 µ8C (grey trace; *n* = 4). **(B,C)** Dose response curves of ERK1 **(B)** and ERK2 **(C)** kinase activity inhibition by KIRA6 (blue trace) and K252a (orange trace) (*n* = 4). **(D–F)** Binding conformations of KIRA6 (orange) and SB203580 (green) in the active site of p38 and the respective docking scores.

We also tested if KIRA6 has direct effects on the activity of p38 and/or ERK. The potent p38 inhibitor SB203580, used as positive control, inhibited p38 kinase activity with an IC_50_ value of 130 nM. Though somewhat less potent than SB203580, KIRA6 also attenuated p38 activity, with an IC_50_ value of about 1 µM (Figure 4A). In contrast, 4 µ8C, another inhibitor of IRE1α, did not affect the kinase activity of p38. On the other hand, KIRA6 did not exhibit any apparent direct effect on the kinase activity of ERK1/2, in contrast to the ERK inhibitor K252a (Figure 4B,C). Furthermore, molecular docking indicates that KIRA6 binds to the active site of p38 (Figure 4D–F). Thus, the docking scores with Autodock Vina for KIRA6 and the potent p38 inhibitor, SB203580, were similar. In addition, KIRA6 and SB203580 were both interacting with key residues of the ATP binding cavity of p38 (Supplementary Figure S4). These results indicate that KIRA6, which belongs to a group of type II kinase inhibitors (Ghosh et al., 2014), potently interacts with p38.

## Discussion

Leukotrienes are pro-inflammatory and immune-modulating lipid mediators that play divergent roles in various pathological conditions and represent important targets for drug development (Haeggström, 2018). The current study presents an unexpected and potent inhibitory effect of KIRA6 on leukotriene biosynthesis in human phagocytes, *viz*. macrophages and neutrophils. The cellular LTB_4_ and cys-LTs production in macrophages was stimulated by two distinct means, i.e., the microbial components LPS and fMLP, or the stress molecule thapsigargin (Figure 1). In both cases, leukotriene production was successfully stimulated, but with large variability from donor to donor, which has been broadly observed in the eicosanoid field and may be due to various factors such as gender and intake of non-steroidal anti-inflammatory drugs.

Inasmuch as KIRA6 blocked leukotriene biosynthesis with equal, or even better potency compared to its action against IRE1α, we initially hypothesized that this arm of the UPR was closely linked to the leukotriene cascade, in analogy with what has been reported for prostaglandin synthesis (Chopra et al., 2019). However, we could not observe any significant effects of KIRA6 on calcium mobilization or key enzymes of the leukotriene cascade, and macrophages made deficient of IRE1α still responded to KIRA6 (Figure 3A,B). Instead, we followed the observation that KIRA6 reduced phosphorylation of cPLA_2_α, which releases AA for leukotriene and prostaglandin biosynthesis. It turned out that in intact cells, the suppression of leukotriene production was associated with reduction of phosphorylated p38 and ERK (Figure 2A–D), established kinases for 5-LOX and cPLA_2_α (Surette et al., 1998; Gijón et al., 2000; Rådmark et al., 2007; Ghosh et al., 2014), and 1 µM KIRA6 was sufficient to mask the inhibitory effects SB203580 and U0126 in leukotriene production (Figure 2E). This suggests that KIRA6 is able to block the p38 and ERK pathways presumably *via* inhibition of mitogen-activated protein kinase kinases (MKKs), for instance MKK3/6 and MKK1/2, respectively, which would in turn reduce p38 and ERK phosphorylation and thereby leukotriene biosynthesis. Moreover, the important decreases of p38 and ERK phosphorylation also predict a reduction of 5-LOX phosphorylation at Ser271 and Ser663, which could contribute to the decreased leukotriene production. However, we were not able to detect endogenous 5-LOX phosphorylation with commercially available antibodies (data not shown), suggesting that 5-LOX phosphorylation, or its inhibition, is not involved in this model.

Besides reducing p38 and ERK phosphorylation, KIRA6 was also found to directly interact with p38 and inhibit its kinase activity. Through molecular docking, KIRA6 was assigned binding affinity to p38 and a potential to enter its ATP-binding domain. Moreover, the inhibitory potency of KIRA6 against p38 kinase activity (IC_50_ = 1 μM, Figure 4A) is close to that reported for the IRE1α-XBP1 UPR activity [IC_50_ = 0.6 µM, (Ghosh et al., 2014)]. It is noteworthy that KIRA6 also exerts inhibitory effects on prostaglandin production by macrophages stimulated with LPS/fMLP, presumably mediated by inhibition of cPLA_2_α (Figure 1E). This provides a second mechanism for KIRA6 in targeting prostaglandin synthesis, apart from the recently described IRE1α-dependent transcriptional regulation of cyclooxygenase-2 and microsomal prostaglandin E synthase-1 (Chopra et al., 2019).

As a potent allosteric inhibitor of IRE1α, KIRA6 has been developed to inhibit IRE1α-mediated cell death, and has been demonstrated to promote cell survival and prevent ER stress-induced cell degeneration *in vivo* (Ghosh et al., 2014). Of note, recent studies have indicated that KIRA6 possesses anti-inflammatory and immune-modulating properties (Chen et al., 2019; Hull-Ryde et al., 2021; Rufo et al., 2022). In light of our results, it is possible that inhibition of the p38/ERK- cPLA_2_α axis of leukotriene synthesis may account for some of these drug effects. Moreover, p38 and ERK participate in a variety of cellular processes by integrating different stress, metabolic, and inflammatory signals suggesting that KIRA6 may interfere with other fundamental cellular processes, yet to be characterized.

In summary, we have uncovered a novel potent action of KIRA6, in which this small molecule interferes with p38 and ERK signaling pathways, leading to inhibition of leukotriene production. While KIRA6 is a promising lead for pharmacological intervention in the IRE1α-XBP1 pathway, further work is required to detail the full pharmacological repertoire of this drug.

## Data Availability

The raw data supporting the conclusions of this article will be made available by the authors, without undue reservation.
