# Supplementary material for: The IRE1α Inhibitor KIRA6 Blocks Leukotriene Biosynthesis in Human Phagocytes
Source: Front Pharmacol. 2022 Mar 22;13:806240. doi: 10.3389/fphar.2022.806240 (PMC8980214; doi:10.3389/fphar.2022.806240)
Supplement: Supplementary file 1 [file DataSheet1.docx]

**Supplementary materials**

**Methods**

**Treatment of KIRA6 with intact cells and cell lysates**

For the experiments with intact cells, KIRA6 (1 µM unless otherwise specified) was added to the cells 30 min before stimulation with LPS/fMLP or thapsigargin. Afterwards, the cells were collected for western blot analysis and the supernatants for lipid mediator analysis.

For the experiments with cell homogenates, 10x10^6^ neutrophils or 3 x 10^6^ differentiated MM6 cells were resuspended in 0.5 mL PBS and kept on ice. Neutrophils and MM6 cells were sonicated 3 x 5 s on ice in the presence of 1 mM EDTA using the VCX 130 Vibra-Cell™ Ultrasonic Liquid Processor. To assess 5-LOX activity, 1 mM ATP was added to the cell homogenates. The samples were pre-incubated for 30 s with 0.2% DMSO or 1 µM KIRA6, followed by incubation with 1 mM CaCl_2_ and 40 µM AA for 10 min. To assess LTC4S activity, cell homogenates were pre-incubated with 0.2% DMSO or 10 µM KIRA6 for 30 s followed by incubation with 5 µM LTA_4_ for 10 min at 37 °C.

**PLA_2_ activity assay**

The PLA_2_ activity assay was carried out based on the manufacturer’s protocol (Cayman Chemicals). The effect of 0-10 µM KIRA6 on the activity of PLA_2_ was assessed with homogenates of differentiated MM6 from 0.5 x 10^6^ cells. Reaction rates are presented as nmol of arachidonoyl thio-PC hydrolyzed by PLA_2_ from one million cells per minute at room temperature.

**Lipid extraction and RP-HPLC analysis of 5-HETE, LTB_4_ and LTC_4_**

Incubations with neutrophils or MM6 homogenates were quenched with 1 mL methanol containing 300 pmol of prostaglandin B_2_ as the internal standard and acidified to pH 3-4 with 15 µL of 3 N HCl. The samples were centrifuged to remove the cell debris at 10 000 x g for 10 min at 4 °C. Supernatants were diluted with 3 volumes of water and applied on the Oasis HLB 3 cc (Waters) solid phase extraction cartridges for further purification based on the manufacturer’s instructions. The eluted samples in methanol were taken to dryness under controlled nitrogen flow with TurboVap LV system (Biotage) and dissolved in 400 µL of methanol:water (1:1) mixture prior to reverse-phase high-performance liquid chromatography (RP-HPLC).

LTC_4_ was analyzed on a 3.9 × 150-mm column (C18; Nova-Pak Waters) by eluting products at a flow rate of 1 mL/min with acetonitrile/methanol/water/acetic acid (30:30:40:0.1; vol/vol) at pH 5.6. For the analysis of 5*S*-HETE and LTB_4_, a mobile phase with acetonitrile/methanol/water/acetic acid (30:36:34:0.1; vol/vol) was used. Absorbance was monitored at 235 nm for 5*S*-HETE (ε ~ 23 000 M^-1^ cm^-1^), 270 nm for LTB_4_ (ε ~ 40 000 M^-1^ cm^-1^) and PGB_2_ (ε ~ 40 000 M^-1^ cm^-1^)_,_ and 280 nm for LTC_4_ (ε ~ 40 000 M^-1^ cm^-1^). Activities in cell homogenates are presented as picomoles of each product per 1 million cells. Results are visualized using the GraphPad Prism program.

**Intracellular calcium assay**

Human monocyte-derived macrophages (hMDMs) were pre-incubated with 1 µM KIRA6 or 0.1% DMSO as the vehicle and loaded with 5 µM Fluo-4 AM (ex/em of Ca^2+^-bound form is 494/506 nm) in parallel at 37 °C for 30 min in a RPMI cell medium. Incubations with 1 µM fMLP were carried out in Krebs buffer, containing 1 mM Ca^2+^ and 1 mM Mg^2+^, or in Ca^2+^-free Krebs buffer with 2 mM EGTA. Baseline was measured for 10 s followed by injection of fMLP using the PolarSTAR Optima plate reader. Recorded fluorescence values were normalized, F/F0, and results are presented using the GraphPad Prism program.

**Supplemental figures**

**fig. S1**


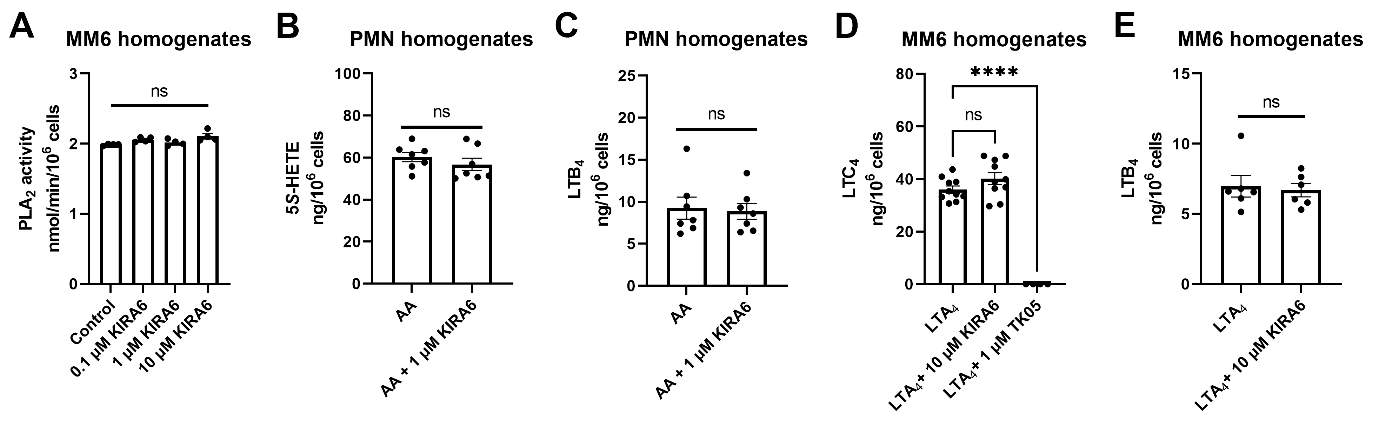


**fig. S1** **KIRA6 has no apparent effect on PLA_2_, 5-LOX, LTC_4_S or LTA_4_H activities.** (A-D) KIRA6 at indicated concentrations was added to homogenates of neutrophils and MM6 to evaluate its direct effect on the activities of PLA_2_ (A) (n=4), 5-LOX (B, C) (n=7), LTC4S (D) (n=10) and LTA4H (E) (n=6).

**fig. S2**


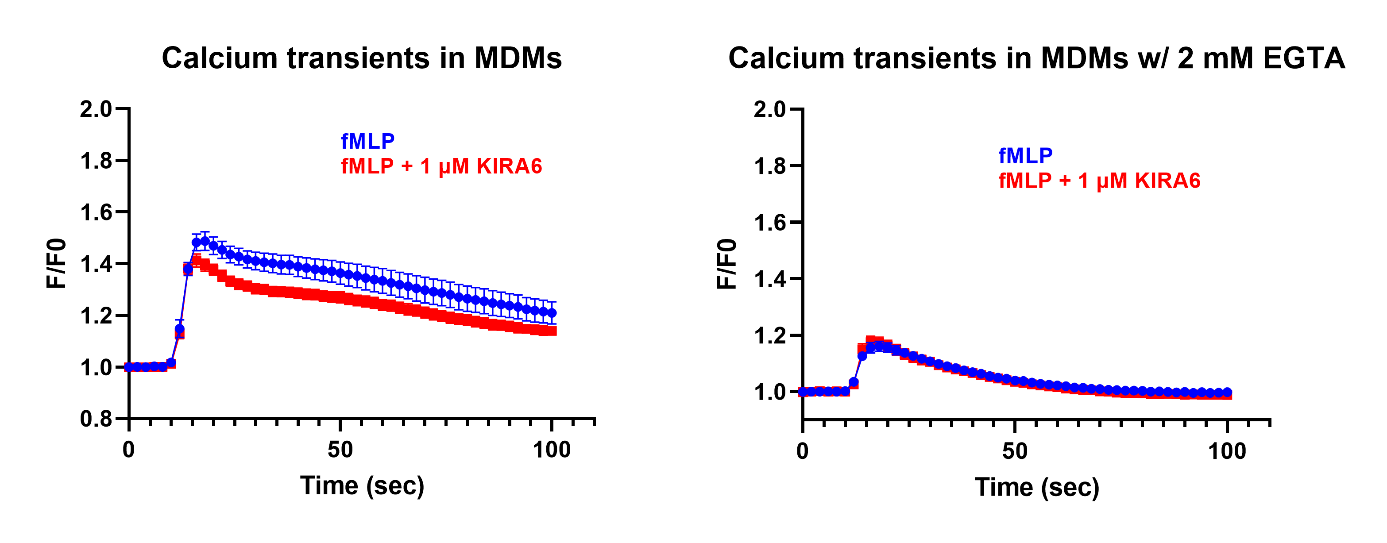


**fig. S2 Lack of apparent effect of KIRA6 on calcium mobilization.** Calcium level was measured in hMDMs, pretreated with 1 µM KIRA6 or vehicle (0.1% DMSO) for 30 min, followed by stimulation with 5 µM fMLP. Left and right panels show results obtained with 1 mM CaCl2 (n=16) or 2 mM EGTA in the culture medium (n=24-28), respectively.

**fig. S3**

**
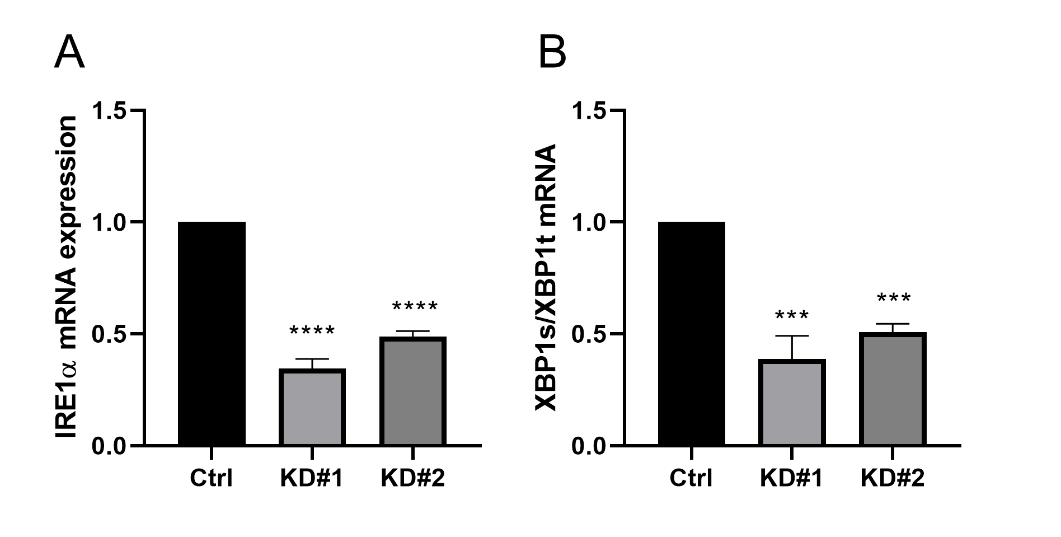
**

**fig. S3 IRE1α mRNA expression and RNase activity in control and IRE1α KD cells.** (A-B) qPCR analysis of IRE1α (A) (n=3), spliced XBP1 (XBP1s) and full-length XBP1 (XBP1t) mRNA (n=3). ****p< 0.001, ****p< 0.0001.*

**fig. S4**

**
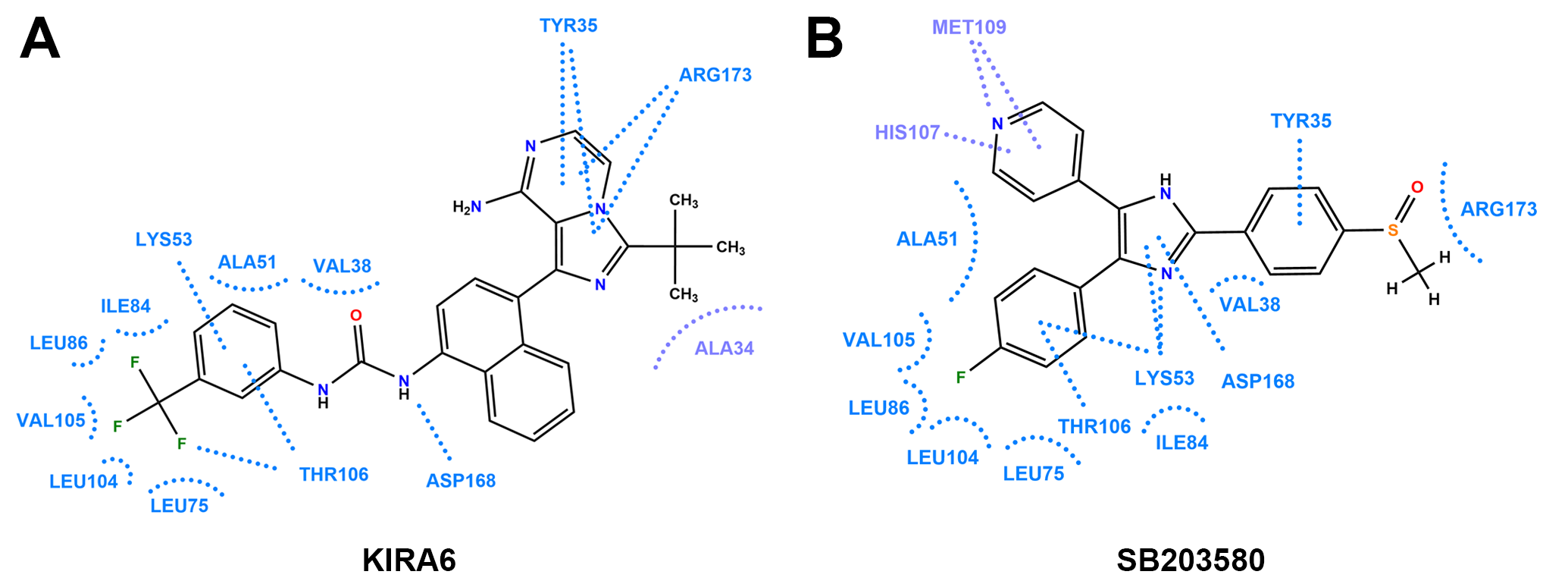
**

**fig. S4 Molecular docking of KIRA6 and SB203580 interactions with p38.** Unique (purple) and common (blue) residues involved in the interaction between p38 and KIRA6 (A) or SB203580 (B).

**Original western blot images**

**Fig. 1F p-cPLA_2_α**

**
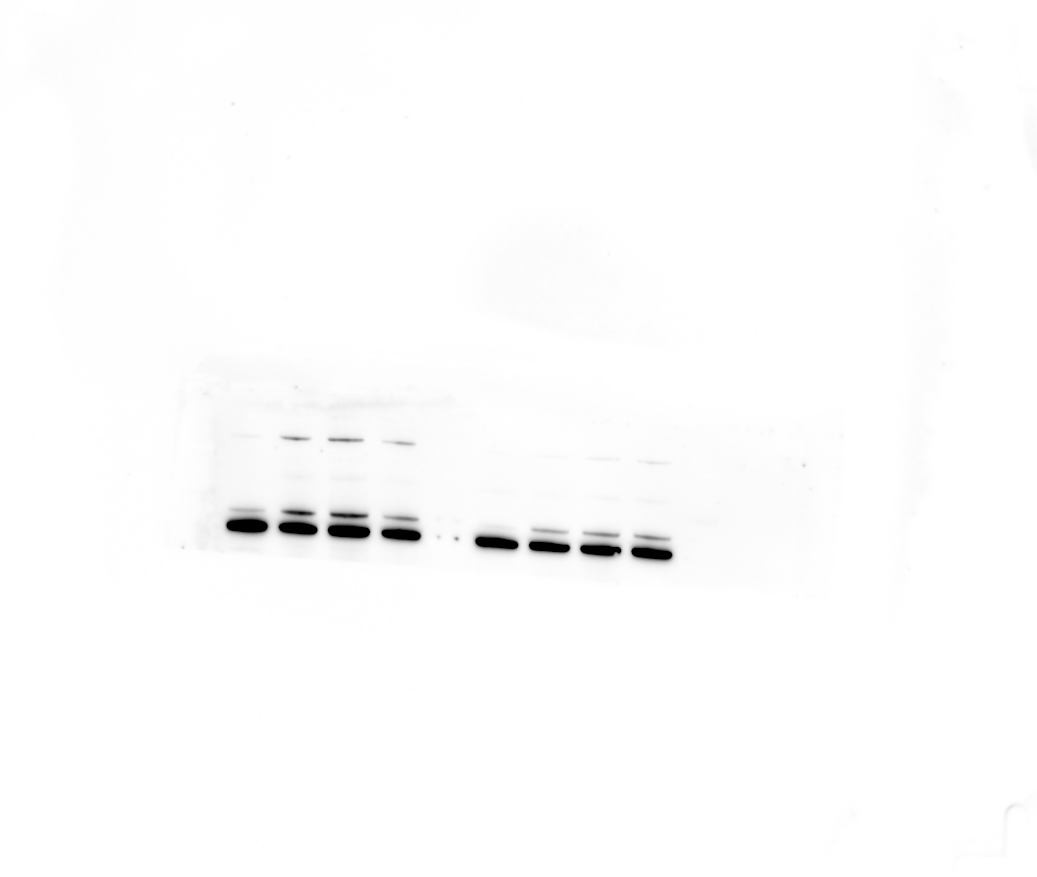
**

**Fig. 1F cPLA_2_α**

**
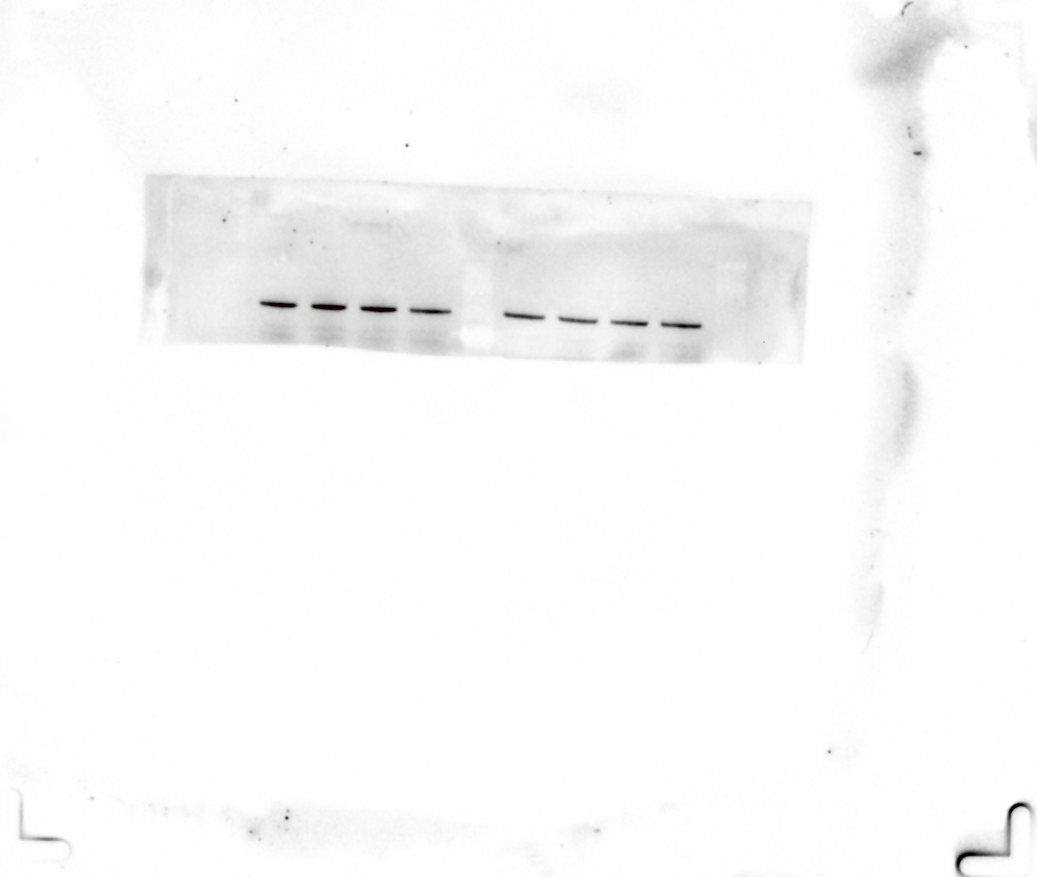
**

**Fig. 1G p-cPLA_2_α**

**
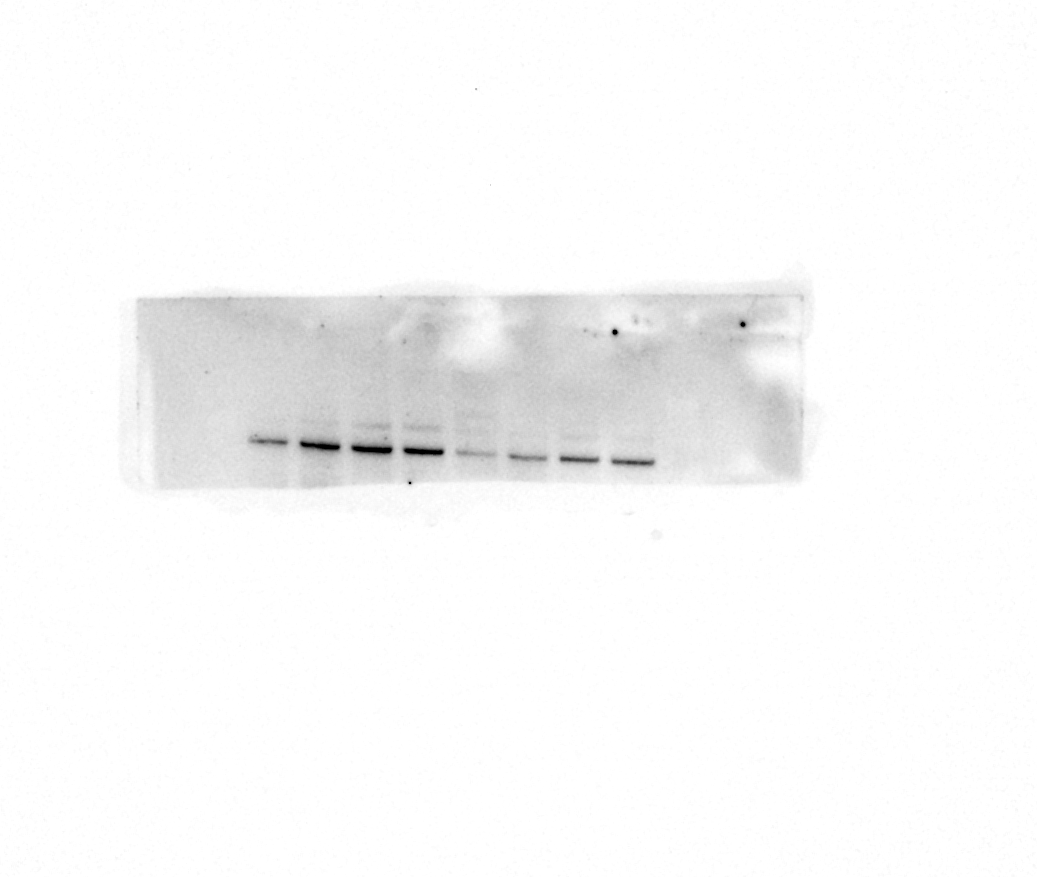
**

**Fig. 1G cPLA_2_α**

**
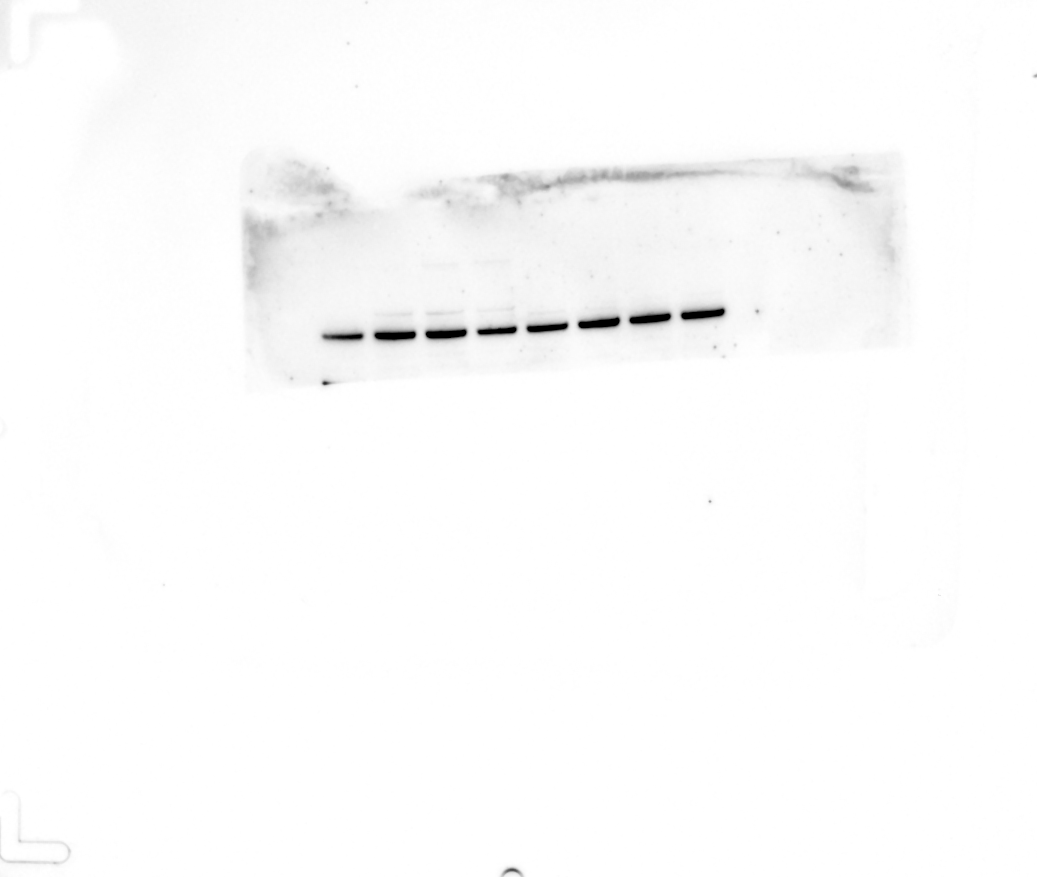
**

**Fig. 2C p-p38**

**
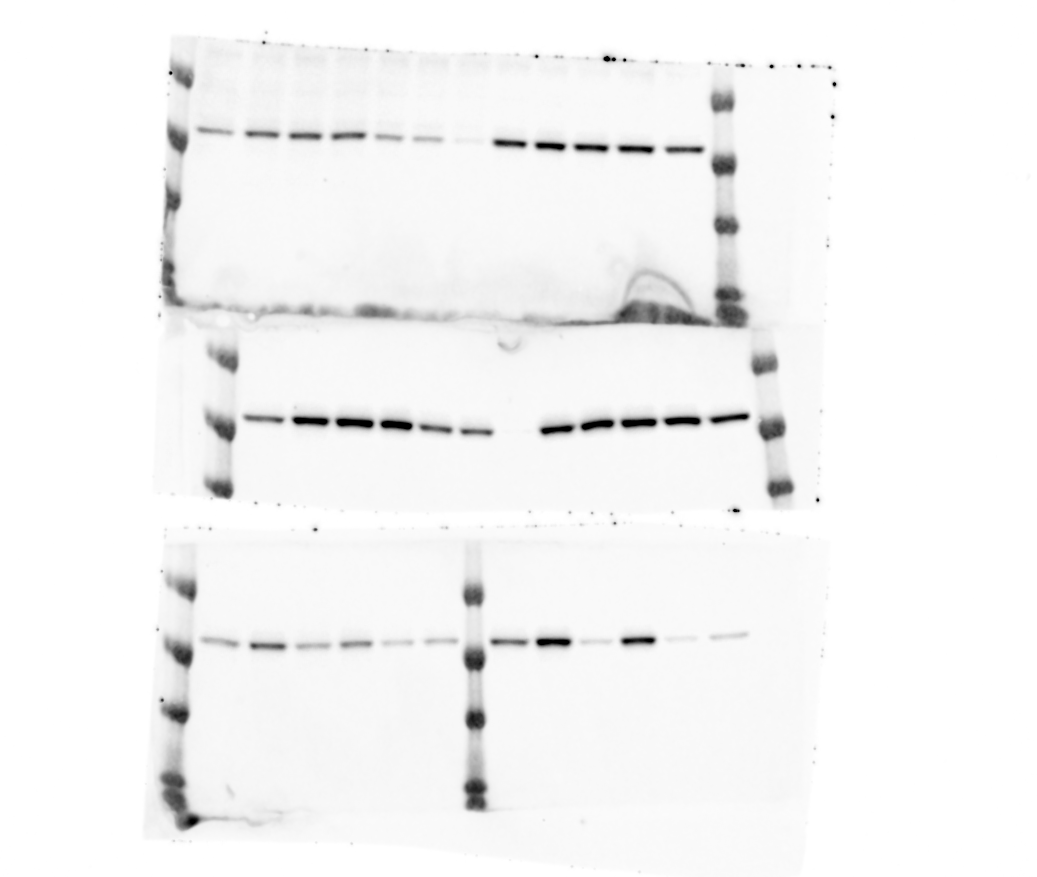
**

**Fig. 2D and Fig. 3C p-p38**

**
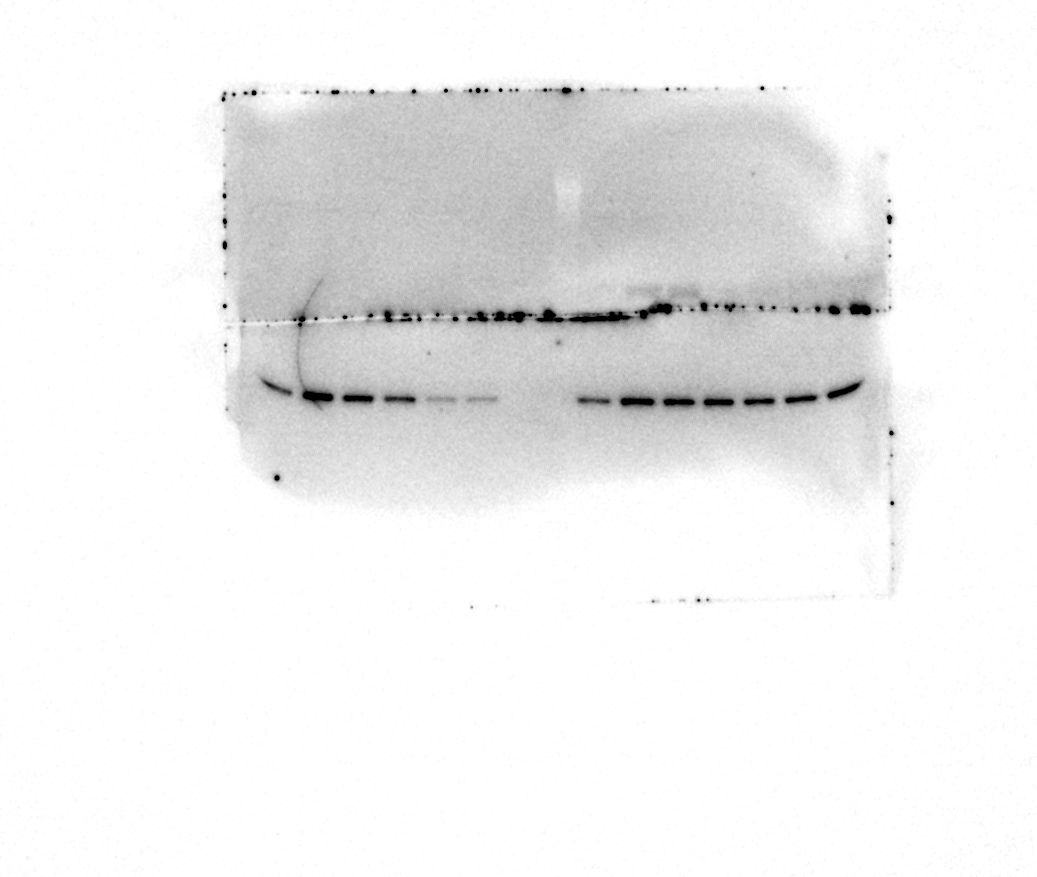
**

**Fig. 3D p-p38**

**
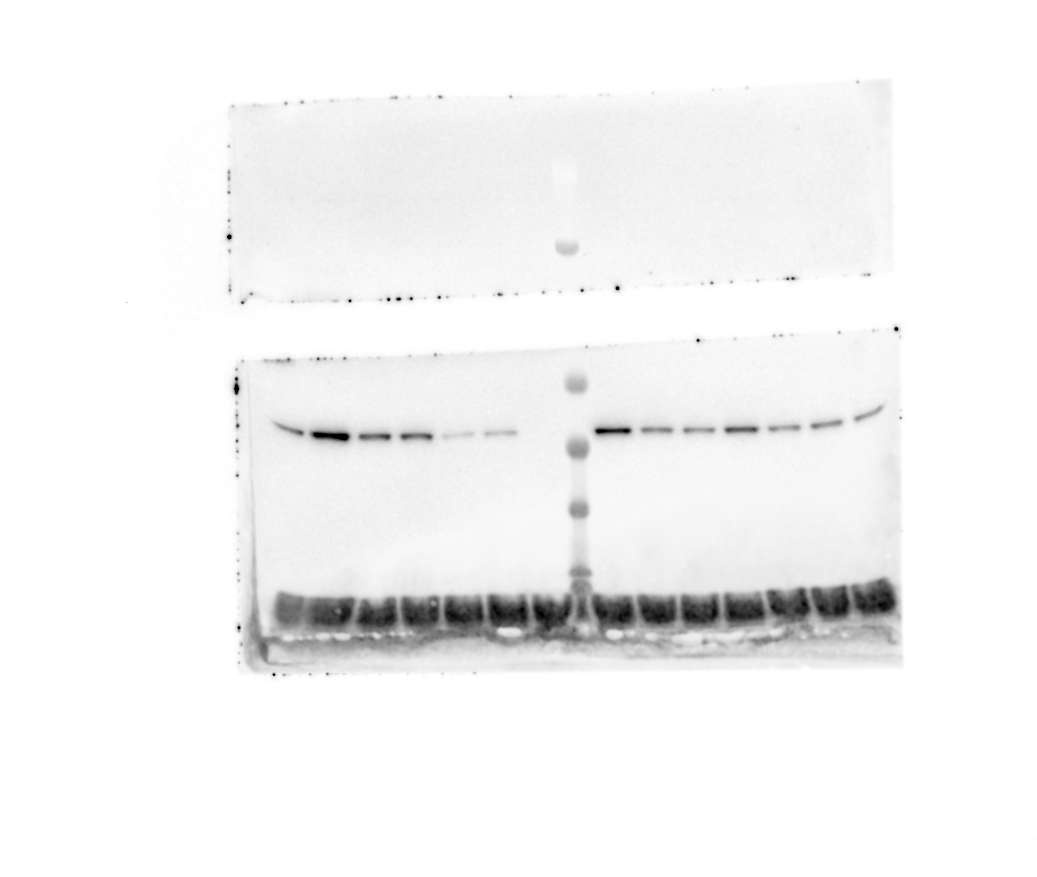
**

**Fig. 2C-D and 3C-D p-ERK**

**
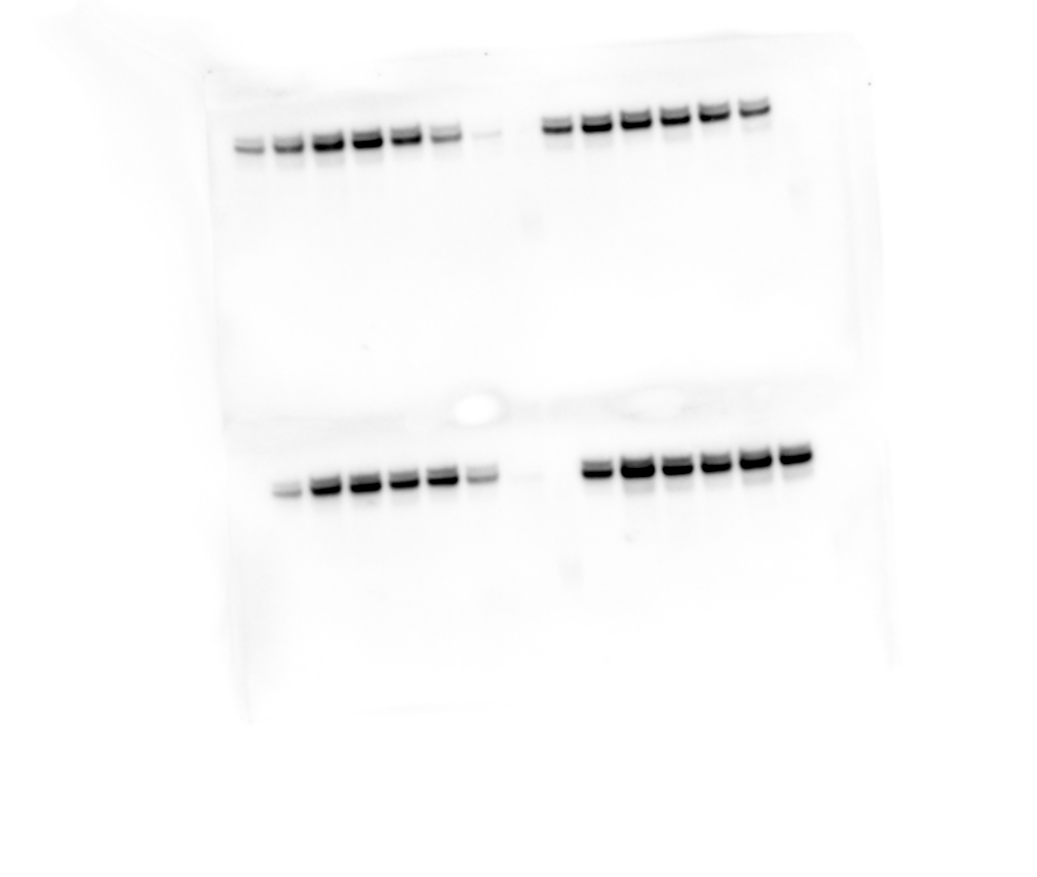
**

**Fig. 2C p-cPLA_2_α**

**
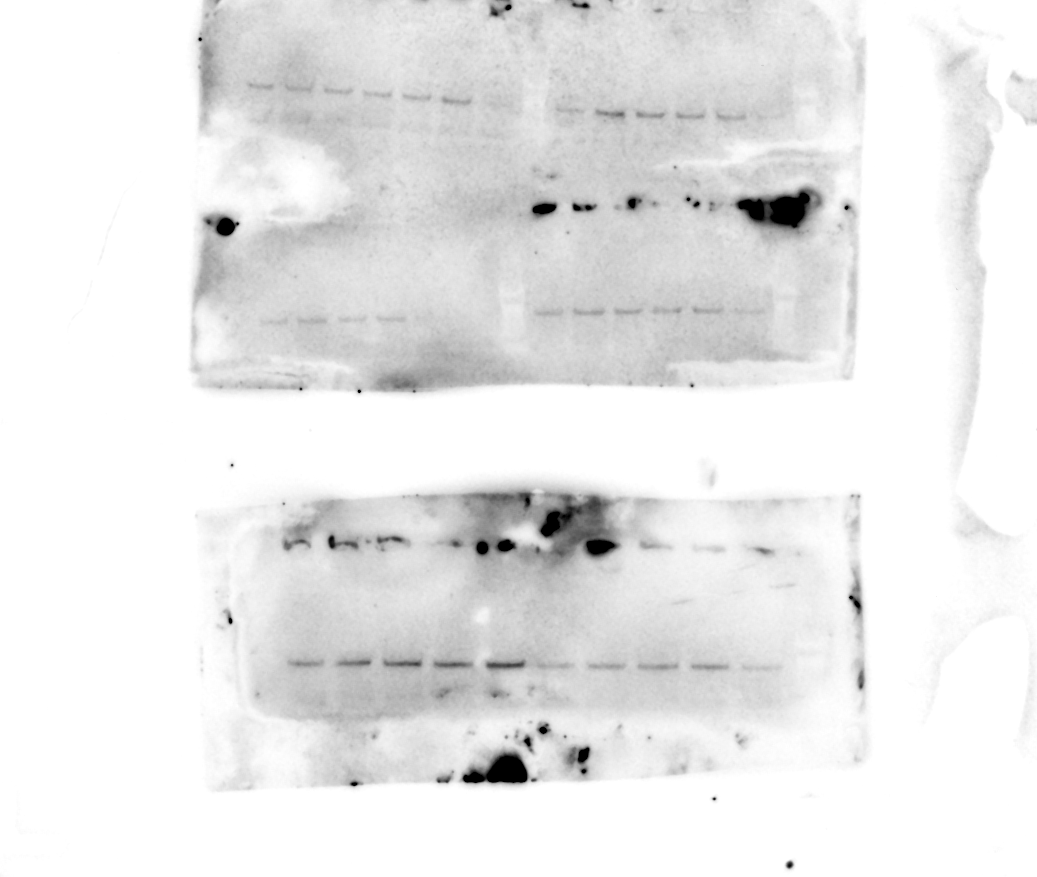
**

**Fig. 2D p- cPLA_2_α**

**
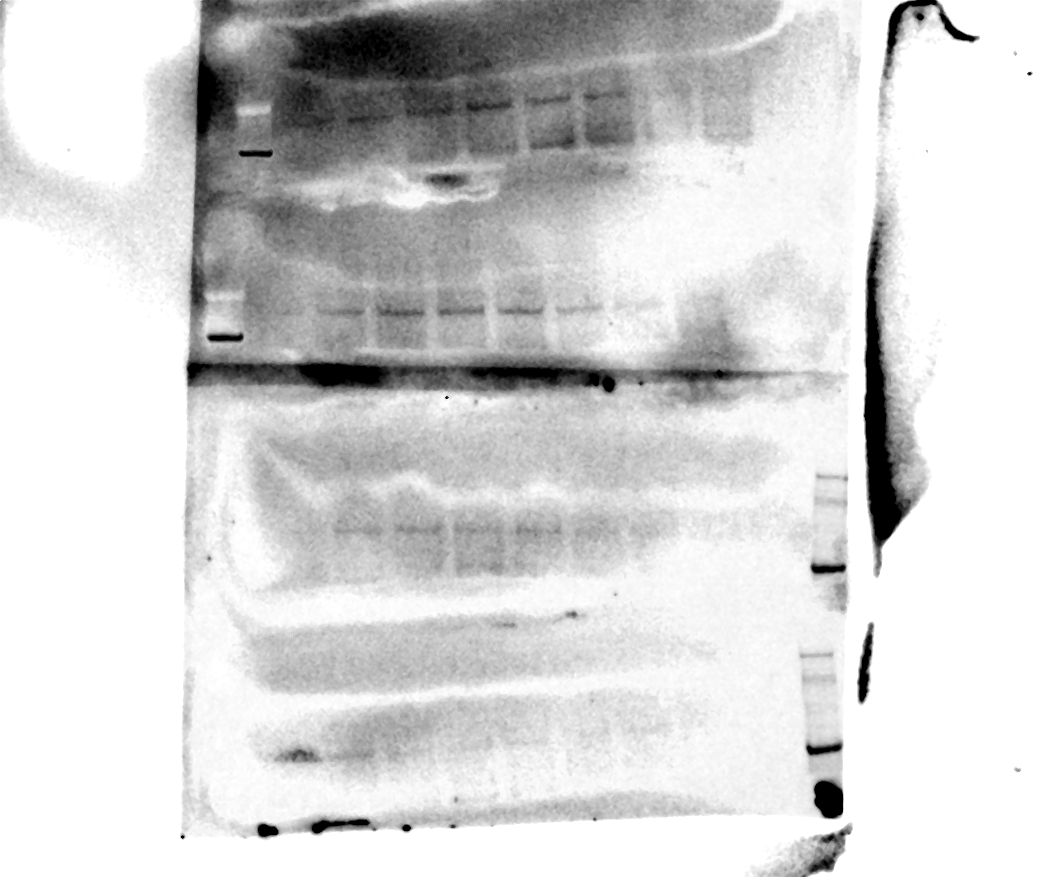
**

**Fig.2C-D and Fig.3C-D β-actin**

**
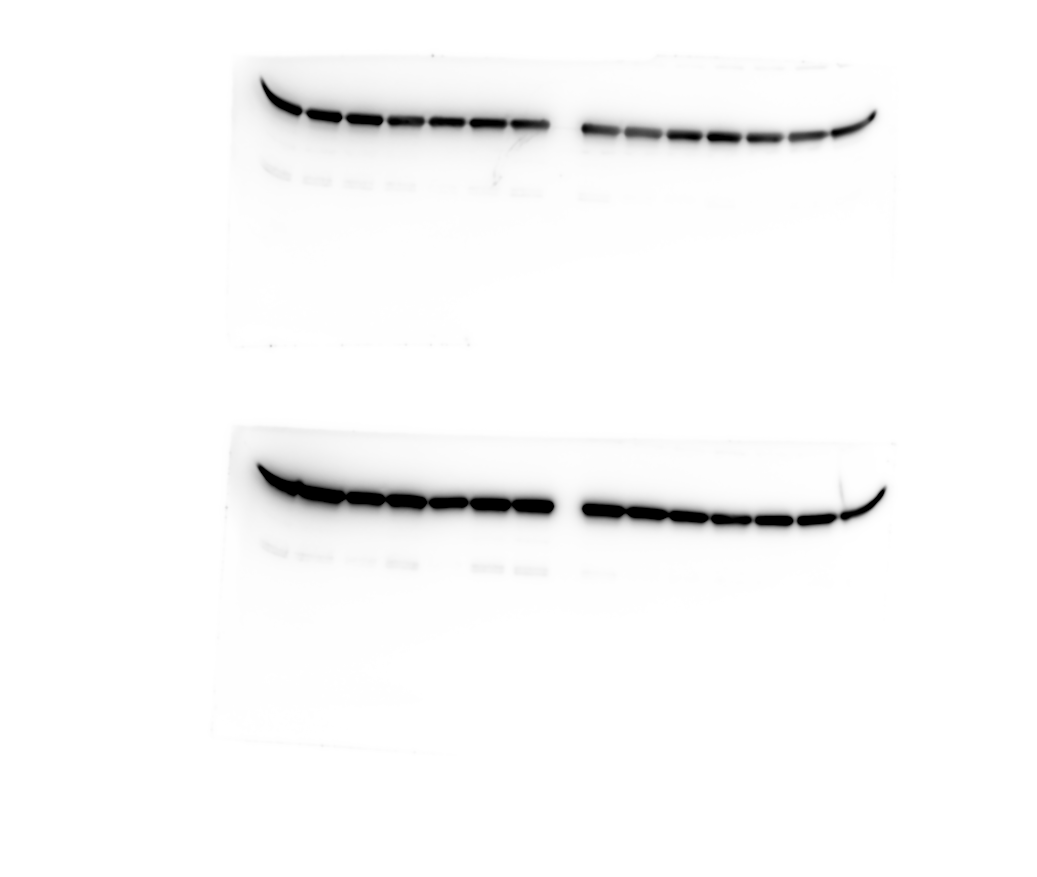
**
